# Mid-Term Results of Distal Femoral Extension and Shortening Osteotomy in Treating Flexed Knee Gait in Children with Cerebral Palsy

**DOI:** 10.3390/children9101427

**Published:** 2022-09-20

**Authors:** Andreas Geisbüsch, Matthias C. M. Klotz, Cornelia Putz, Tobias Renkawitz, Axel Horsch

**Affiliations:** 1 Department of Orthopedics, Heidelberg University Hospital, 69118 Heidelberg, Germany; 2 Marienkrankenhaus Soest, Orthopedics and Trauma Surgery, 59594 Soest, Germany

**Keywords:** distal femoral extension, shortening osteotomy, DEFSO, cerebral palsy

## Abstract

**Background:** Distal femoral extension and shortening osteotomy (DFESO) seems to be an effective method for the treatment of flexed knee gait in children with cerebral palsy. Nevertheless, studies investigating the mid- and long-term outcomes after such procedures are lacking in the literature. Therefore, the purpose of this study was to assess the mid-term outcomes regarding sagittal plane kinematics of the knee after DFESO with or without concomitant patella advancement. Furthermore, an evaluation of the postoperative course and possible recurrence of flexed knee gait was planned. **Methods:** In a prospective observational study, 19 patients (28 limbs; mean age 11.8 years (6.7–16.0 years)) were examined using 3-D gait analysis and clinical exam before (E_0_) and at a mean of 38 months (E_2_: 24–55 months) after surgery. Fifteen patients (22 limbs) had an additional first postoperative gait analysis (E_1_) after a mean of 14 (10–20) months after surgery. In these patients, the postoperative changes between the short-term and mid-term gait analyses were evaluated. **Results:** DFESO led to a significant decrease in flexed knee gait with an improvement in sagittal plane kinematics during the stance phase. In addition, a slightly increased anterior pelvic tilt was observed at E_1_, and we found a tendency towards stiff knee gait with a decrease in mean knee flexion in swing at E_2_. **Conclusions:** DFESO led to a significant improvement in flexed knee gait in children with cerebral palsy. The therapeutic effect seems to be lasting on mid-term follow-up with a slight overall tendency to recurrence.

## 1. Introduction

Flexed knee gait is one of the most common gait patterns in children with cerebral palsy. It impairs gait and leads to increased energy consumption and restriction of ambulation [1]. Apart from the restrictions in gait and self-independence, flexed knee gait may cause anterior knee pain due to increased pressure on the anterior part of the knee and chondromalacia [2]. One of the main pathologic findings in patients showing flexed knee gait is an abnormal tone and/or shortening of the hamstrings [1]. Therefore, lengthening of the hamstrings as a part of single-event multilevel surgery (SEMLS) has been successfully performed for this condition for many years [3,4,5,6,7]. Despite good short- and mid-term results after hamstring lengthening, deterioration in gait and recurrence of flexed knee gait have been reported [8]. A further side effect of hamstring lengthening is an increase in anterior pelvic tilt, which might mediate the recurrence of flexed knee gait [9,10,11]. Attempts to convert the muscle group into a monoarticular function also showed no improvement in pelvic inclination [8,12]. Over the past years, an increasing number of studies reported the results of treatment of flexed knee gait through distal femoral extension osteotomy with or without shortening of the femur [13,14,15,16,17]. The procedure showed promising short-term results with excellent correction of flexed knee gait [13,18,19]. However, the correction also seems to have an impact on the anterior pelvic inclination [19]. This increase in pelvic tilt might be due to a decreased stabilization of the pelvis due to the relative and functional lengthening of the hamstrings [20]. In addition to that, the tilt of the pelvis also depends on the muscle tone of the rectus femoris, which acts as an antagonist to the hamstrings [8,16]. Nevertheless, in a previous study, Klotz et al. found that simultaneous recession of the rectus femoris could not prevent an increase in anterior pelvic tilt after distal femoral extension and shortening osteotomy [19]. Reports on mid-term and long-term results of DFESO are still scarce [21,22].

Boyer et al. reported the first long-term results of DFESO with patella tendon advancement in crouch gait. They reported a superior effect of DFESO plus patella tendon advancement compared to other methods and significantly less recurrence of flexed knee gait after a period of 13 years [21]. Kuchen et al. reported maintained improvements of sagittal gait kinematics nine years after patellar tendon shortening combined with DFEO [22].

The objective of this study was to assess the mid-term results after distal femoral extension (plus shortening) osteotomy (DFE(S)O) with or without concomitant patella advancement (PA). We sought to evaluate the impact of DFE(S)O on sagittal plane knee joint kinematics, pelvic tilt and general gait quality in patients with cerebral palsy. We hypothesized that DFS(E)O improves sagittal plane gait kinematics, and the changes are maintained until mid-term follow-up (24–60 month). We further hypothesized that DFS(E)O increases pelvic tilt.

## 2. Methods

### 2.1. Study Design and Patient Selection

This monocentric prospective observational study evaluates the results of a study population that was treated with DFESO as a part of SEMLS in the Orthopaedic Department of the University Hospital Heidelberg. All included patients had (1) a preoperative three-dimensional (3-D) gait analysis (E_0_) prior to the surgical intervention, (2) a GMFCS level I–III (ambulatory walkers), (3) flexed knee gait (defined as a minimum knee flexion during stance of more than one standard deviation compared to an age-matched control group), (4) a bilateral spastic cerebral palsy (BSCP) as the underlying pathology and were (5) between 6 and 16 years old. Patients who underwent surgical treatment or Botulinum toxin injections within the last six months prior to surgery were not considered (exclusion criteria). Patients were considered for mid-term evaluation if postoperative three-dimensional (3-D) gait analysis data were available between 24 and 60 months after surgery (E_2_). If an additional 3-D gait analysis was available postoperatively within 18 months, we defined the results as first postoperative control (E_1_) to evaluate possible changes between the short-term and mid-term outcomes of these patients. Furthermore, we formed two subgroups of patients that had or had not undergone simultaneous patella tendon advancement in context with the DFESO.

The local ethics committee approved the study.

### 2.2. Operative Procedure

The indication for DFESO was set in patients with a flexed knee gait and an aberration of more than one standard deviation from the normal collective in the minimum knee flexion during stance in the 3-D gait analysis (less than eight degrees of minimum knee flexion). The amount of correction was defined with the clinical examination of the flexion contracture intraoperatively [19]. The operation was performed as described by Novacheck et al. and Brunner et al. [14,23], with a standard lateral approach to the distal femur. A ventrally based trapezoid-shaped osteotomy of the distal femur was performed, and after correction of the deformity, it was fixed with a plate. The fixation was performed with blade plates (Implantcast, Buxtehude, Germany) in 15 cases, locking plates by Königssee (Königsee Implantate, Allendorf, Germany) in 3 cases, and locking plates of DePuySynthes (DePuySynthes, Westchester, NY, USA) in 1 case. An additional patella advancement was performed in the presence of patella alta. As the surgery was performed within other SELMS procedures, additional surgical interventions were performed if necessary.

### 2.3. Postoperative Treatment

In accordance with the previously described regime of postoperative treatment after DFESO [19], patients were primarily immobilized in plaster casts with a rigid connection between the casts to ensure an external rotation of the leg of 10–15° degrees. A slight knee flexion of 10° was applied. Early physiotherapeutic treatment was administered in all patients with passive and active mobilization of both the hips and knee joint starting at the first day after surgery. Knee flexion was limited to 40° for two weeks and 60° between the second and fourth postoperative week. All children were treated by a team of orthopedic surgeons in cooperation with physiotherapists and nursing staff with specialized training. All children were referred to rehabilitation institutions specializing in neuromuscular diseases in children after the consolidation of the osteotomy was achieved and weight-bearing was permitted.

### 2.4. Gait Analysis

Gait analysis and clinical examination were undertaken by physiotherapists and a study nurse specialized in pediatric neuro-developmental therapy. All patients were able to walk freely and asked to walk barefoot on a seven-meter-long walkway at a self-selected speed. Skin-mounted markers were applied to the bony landmarks according to a standard protocol. A Vicon camera system (Vicon^®^, Oxford Metrics, Oxford, UK) and two piezoelectric force plates (Kistler^®^, Winterthur, Switzerland) were used, and joint kinematics were calculated with Vicon-Workstation^®^ (Oxford Metrics, Oxford, UK) according to the protocol of Kabada et al. [24]. Kinematic data were acquired for the knee and pelvis. The physical examination recorded the range of motion and popliteal angle.

### 2.5. Data Acquisition and Evaluation

**Figure 1:**
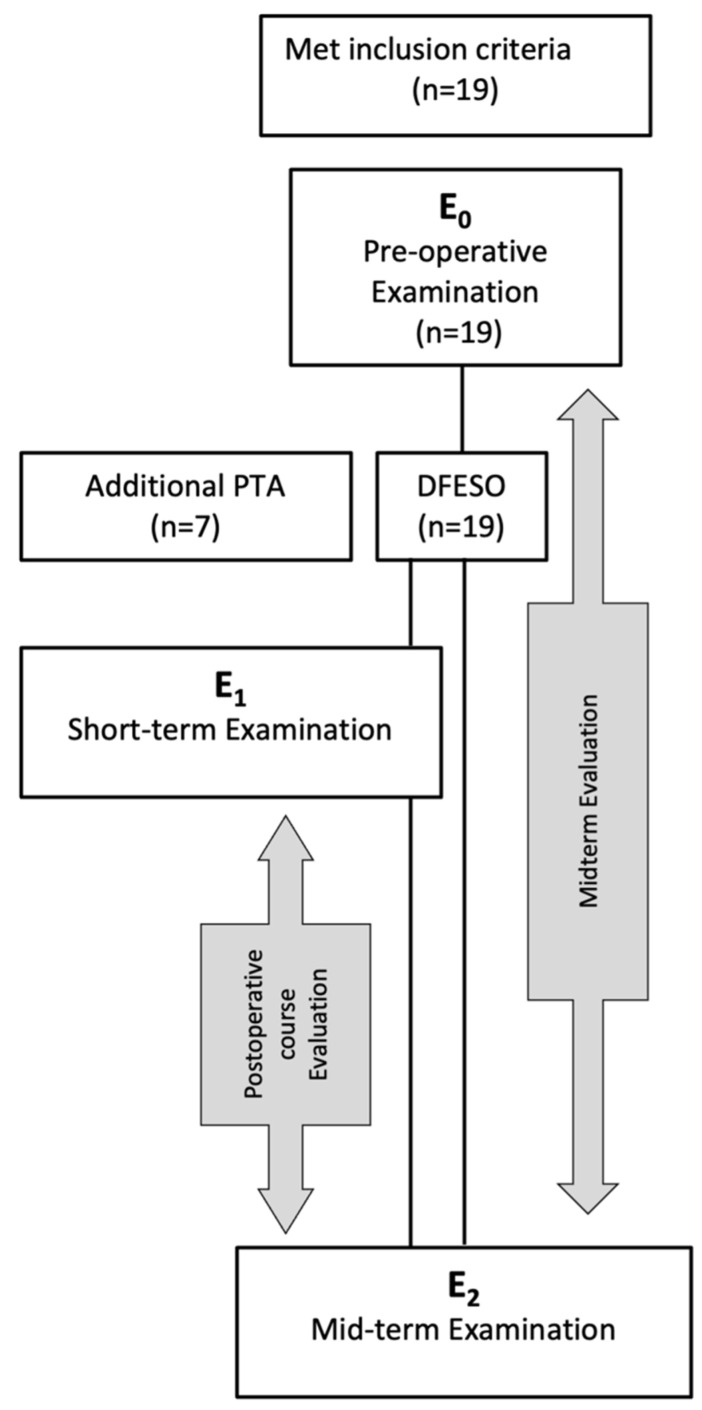
Overview of the different time points of evaluation. E_0_: preoperative, E_1_: short-term examination 6–18 month postoperative; E_2_: mid-term examination 24–60 month postoperative.

Nineteen patients (28 limbs) met the inclusion criteria mentioned above and were selected from the motion lab database (Figure 1). All patients had a preoperative analysis (E_0_) and received DFESO. Of those, seven children were treated with additional patella tendon advancement (PTA). Table 1 gives an overview of the concomitant procedures performed during the SEMLS procedure. These included bony and soft tissue reconstructions of the hip, or transposition of the rectus femoris, lengthening of the hamstrings, correction of equinus foot through calf muscle lengthening and correction of the hind foot. Fifteen patients (22 limbs) had a three-dimensional gait analysis at short-term follow-up (E_1_; up to 18 months after DFESO). All 19 patients were followed for the mid-term evaluation between 24 and 60 months after surgery (E_2_). As a first step, we calculated the results of patients between E_0_ and E_2_ to determine the mid-term results after DFESO. After that, we analyzed the postoperative course by evaluating the results of patients that had an additional short-term examination (E_1_). A further subgroup analysis was performed for patients that had undergone DFESO plus PTA. The data were analyzed using SPSS statistics (IBM SPSS Statistics 19, IBM, Ehningen, Germany). To evaluate the normal distribution of the data, the Shapiro–Wilk test was applied. The data were further evaluated using either the Student’s *t*-test in the case of normal distribution of the data or the Wilcoxon signed-rank test if data were not normally distributed. Statistical significance was set at a *p*-value < 0.05 for Student’s *t*-Test and 0.025 for Wilcoxon signed-rank test. If patients had repeated postoperative gait analysis, an ANOVA for repeated measures with Bonferroni correction or Friedman’s test with post hoc analysis were used. To compare the subgroup that received PTA to the non-PTA group Mann–Whitney U-Test was applied.

## 3. Results

Overall, 19 patients (28 limbs) could be included in the study and evaluation. The mean age at surgery was 11.8 years (6.7–16.0). All patients had a preoperative gait analysis at a mean of 3.7 (0.5–9) months prior to surgery. The mean mid-term follow-up time (E_2_) was 38 (24–55) months. Fifteen patients (22 limbs) had an additional first postoperative gait analysis (E_1_) after a mean of 14 (10–20) months after surgery. There was no evidence of non-union or implant failure. All patients were ambulatory walkers, including eight patients with a GMFCS level III, eleven patients with a GMFCS level II and only one patient with a GMFCS level I. Kinematic data of the knee and pelvis, as well as physical examination data, were available in all patients. Apart from the kinematic data of the pelvis, the statistical testing showed normal distribution of the values.

### 3.1. Kinematic Results

**Figure 2:**
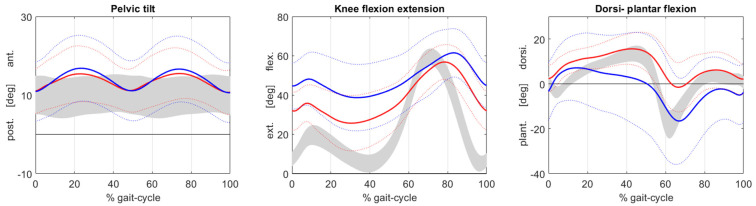
Sagittal plane kinematic graphs illustrating the pelvic tilt, knee flexion and extension and ankle dorsi- and plantar flexion during preoperative (E_0_: blue line) and mid-term (E_2_: red line) gait analysis. The grey areas represent gait data of an age-matched control group. The *x*-axis represents gait cycle in percent; *y*-axis marks the range of motion in degrees. Solid lines represent average values and dashed lines one standard deviation.

There was a significant change in all sagittal plane kinematic values of the knee at E_2_ in comparison to the preoperative values (min. and mean knee flexion in stance, min. knee flexion at initial contact, ROM over 100% GC, mean knee flexion over 100% GC, mean and peak knee flexion in swing) (Table 2 and Figure 2). The mean minimum knee flexion in stance was 38° (SD17°) at E_0_ in accordance with the severe flexed knee gait preoperatively. There was a significant improvement in the minimum knee flexion in stance of 16° (SD 17°) from E_0_ to E_2_ (*p* < 0.001). The mean knee flexion in stance also improved from 46° (SD 19°) preoperatively to 33° (12°) postoperatively (E_2_) (*p* = 0.001), while the range of motion showed an overall improvement of almost 10° from E_0_ to E_2_. We found a significant change in the mean knee flexion over the entire gait cycle from 50° (SD 15°) at E_0_ to 37° (SD 9°) at E_2_ (*p* < 0.001). In contrast, the mean knee flexion in swing showed a decline from 65° (SD12°) to 58° (8°) (*p* = 0.008). The same effect was seen for peak knee flexion in swing with a significant overall decrease of 7° (*p* = 0.008). The individual case analysis showed an improvement in the min. knee flexion in stance of more than 80% in one case, 60–80% in six cases, 40–60% in five cases, 20–40% in six cases and 10–20% in six cases. Four patients (six limbs) showed a worse minimum knee flexion in stance at E_2_ than preoperatively.

The sagittal plane kinematic data of the pelvis revealed a change in the maximum anterior pelvic tilt from 18° (SD 9°) preoperatively to 17° (SD 7°) at E_2_. The mean anterior pelvic tilt changed from 14° (SD 8°) to 13° (SD 6°). Both changes were not significant in the analysis (*p* = 0.72 and 0.58).

### 3.2. Clinical Examination

The preoperative clinical examination revealed a flexion contracture of 15° (SD 7°), which improved by 12° at E_2_ (*p* < 0.001). The mean popliteal angle was 42° (SD 23°) at E_0_ and showed a significant change towards a full extension of 8° (SD 12°) at E_2_ (*p* = 0.01).

### 3.3. Changes in the Postoperative Course (E_1_ to E_2_)

Min. knee flexion in stance changed from 21° (SD 13°) to 23° (SD 13°) from E_1_ to E_2_ (*p* > 0.05). The mean knee flexion in stance and knee flexion at initial contact showed a decline in the postoperative period–mean knee flexion in stance of 5° and knee flexion at initial contact of 3°. The changes were non-significant for both parameters (*p* = 0.055 and *p* = 0.06) (Table 3). Peak knee flexion in swing increased by 5.6° during the postoperative course. The clinical examination revealed an increase in the popliteal angle from 30° (0°–70°) to 34° (15°–60°) from the postoperative to mid-term evaluation. The flexion contracture as measured in the clinical examination changed from 3° (0°–15°) postoperatively to 5° (0–20°) at E_2_. This change proved to be non-significant in the post hoc analysis with Bonferroni correction (*p* > 0.05). The mean pelvic tilt showed a non-significant trend toward preoperative values in the mid-term evaluation (E_1_: 15° (SD9°) and E_2_: 13° (6°)). The same tendency was observed for the maximum pelvic tilt.

### 3.4. Patella Tendon Advancement

There was no significant difference in the severity of flexed knee gait between the PTA and non-PTA groups at E_0_ (min. knee flexion in stance: *p* = 0.48, min. knee flexion at initial contact: *p* = 0.17, mean knee flexion: *p* = 0.64). Patients with additional patella tendon advancement (n = 9) showed no significant difference from the non-PTA group (n = 19) in minimum knee flexion in stance, min. knee flexion at initial contact or peak flexion in swing at the mid-term control (E_2_) (*p* = 0.73, 0.15 and 0.96). There was also no evidence of a significant difference in maximum pelvic tilt between the groups at E_2_.

## 4. Discussion

Flexed knee gait is one of the most common gait patterns in children and adults with cerebral palsy [1]. It leads to relevant functional restrictions during gait impairing independence and quality of life [25]. Several techniques to improve flexed knee gait have been introduced in the past. Soft tissue procedures, especially lengthening of the hamstrings, have proven to be effective in short-term analysis [3,4,9,26]. However, varying results following hamstring lengthening in long-term evaluation have been published [7,8]. DFESO is an alternative treatment method to address flexed knee gait and fixed flexion deformity. Early reports of the positive effects of the procedure on sagittal plane kinematics of the knee were corroborated by various authors [13,14,15,16,17,18]. Nonetheless, few studies have investigated the mid- and long-term effects of DFSEO on crouch gait [21,22]. De Morais Filho et al. reported early results with an improvement in sagittal knee joint kinematics after DFEO after 24 months [15] and further evaluated the procedure in combination with patella tendon advancement with a follow-up of 34 months [16]. Sossai et al. found an improvement in minimum knee extension in stance by 22° after 22-month follow-up [17]. The first long-term data were published by Boyer et al. in 2018 comparing the results of DFEO in combination with PTA to non-surgical treatment [21]. Kuchen et al. evaluated the postoperative course in twelve patients that were treated with SELMS due to flexed knee gait. The authors found a maintained improvement of gait kinematics and walking speed after a nine-year follow-up [22].

In this study, we found an improvement in knee kinematics in patients that underwent DFESO with and without PTA, both in short-term and mid-term evaluation. The good results of previous work concerning the short-term outcome [19] could be confirmed with the present data. Furthermore, a lasting improvement in knee kinematics could be measured in the mid-term evaluation after a mean of 38 months. This was also reflected by a substantial improvement in the GDI at mid-term, indicating an overall lasting improvement in gait quality, affirming our first hypothesis. These findings are in keeping with other studies that found lasting improvements after flexed knee gait correction [21,22]. Nonetheless, a tendency towards preoperative flexed knee gait was evident. There was deterioration in all sagittal plane kinematic values of the knee between E_1_ (10–24 months postoperative) and E_2_ (24–60 months postoperative), but only the changes in mean knee flexion in stance were statistically significant. However, the mean kinematic values of the presented study must be interpreted with caution. While we saw an overall improvement of the kinematic values at E_2_, eleven patients showed a minimum knee flexion in stance more than two SD (18°) worse than the control group, suggesting that these patients still walked in crouch gait. Boyer et al. reported a persisting crouch gait in 37% of patients treated with DFEO and PTA in their long-term analysis after eight years [21]. The mid-term results of our study show an even higher percentage of persisting knee flexion gait. In contrast, Kuchen et al. published consistent improvement in gait during follow-up without relevant changes between postoperative, mid- and long-term evaluation [22]. Among other factors, a tightness of the hamstrings during growth leading to recurrence of flexed knee gait must be considered [8]. However, the sample size in this study is too small to allow an evaluation of the influence of age at surgery on the mid-term outcome of DFE(S)O. In addition, the follow-up time of 38 months might not have been long enough for this effect to develop. Numerous concomitant procedures were performed in context with DFESO. Hamstring lengthening was performed in five cases and may have influenced the results of the correction. Healy et al. found that hamstring lengthening is rarely necessary in patients with crouch gait and treatment with DFEOS, as the bony correction itself restored hamstring length and improved hamstring velocity [20]. De Morais Filho et al. reported a larger reduction in knee flexion in stance when additional hamstring lengthening is performed in combination with DFEO and patella tendon shortening, but also noted an increased anterior pelvic tilt in these patients [16]. The patients that received additional hamstring lengthening in our study showed less improvement in knee extension at E_2_, but due to the small number of cases the relevance of this finding is questionable. Bony foot corrections to address lever arm dysfunction were also among the most frequent additional procedures. They were often accompanied by equinus correction through calf muscle lengthening in the presence of persisting intraoperative equinus deformity. In these cases, overcorrection, which can lead to calcaneal gait and therefore mediate recurrence of crouch gait, must be carefully avoided [27]. Although we found four patients with increased ankle dorsiflexion postoperatively, only one patient demonstrated calcaneal gait at E2 in combination with a deterioration in sagittal plane knee kinematics.

In a previous study evaluating the short-term outcome of DFESO, a significant increase in the anterior pelvic tilt with possible clinical impact was found [19], corroborating the findings of other authors [13,15] reporting increased anterior pelvic tilt after DFESO [16,28]. In the present study, both mean and maximum pelvic tilt were nearly identical in the preoperative and the mid-term evaluation. A slight increase in the anterior pelvic tilt was observed at E_1,_ but this tendency was not statistically significant, and the increased tilt vanished in the mid-term control thus dismissing our second hypothesis. However, the increase in pelvic tilt in the immediate postoperative cause might be one of the reasons for the recurrence of flexed knee gait over time, as it can be compensated by increased flexion of the knee [8]. An increased muscle tone in the rectus femoris and psoas muscle may also influence the anterior pelvic tilt, as the balance between hip extension and hip flexion is altered [13,16,29,30]. Böhm et al. identified an increased muscle tone of the rectus femoris as one of the key factors in increased pelvic tilt after correction of crouch gait [29]. The effect was also discussed by de Morais Filho et al., and the authors noted that none of their patients had received an additional rectus femoris transfer [16]. A substantial number of patients in our study received a transposition of the rectus femoris and recession of the psoas. This might explain why anterior pelvic tilt was not as evident in comparison to other studies. In contrast to our data, Stout et al. reported no influence of rectus femoris transfer or psoas lengthening on anterior pelvic tilt in patients that received DFEO and PTA [13]. Furthermore, we observed a slight decrease in the mean and peak knee flexion during swing phase, indicating that DFESO led to a tendency to stiff knee gait, corroborating the results of other authors [17,28]. Sossai et al. reported a decrease in peak knee flexion in swing after patella tendon shortening, although not all patients simultaneously underwent supracondylar extension osteotomy [17]. Park et al. observed a similar effect after a 24-month follow-up when knee flexion deformity is addressed with a femoral shortening osteotomy [28]. During the postoperative course we observed an improvement in the peak knee flexion in swing, although the tendency did not fully compensate for the postoperative loss. An increased walking speed could also have led to a reduced knee flexion in swing. The observed increase in walking speed could therefore have been at least partially responsible for the loss of knee flexion in swing.

In 2018, Broyer et al. published the first long-term data on patients that underwent DFESO + PTA [21]. The authors compared the results of the procedure to patients with crouch gait who did not undergo surgical intervention. They found a significant improvement in static and functional outcome parameters in the DFESO + PTA group after eight years. At the same time, there was a trend towards a slight deterioration in the initial correction. In the present study, a similar effect was found at mid-term evaluation.

DFESO can be performed in combination with PTA in the presence of patella alta. Although differing reports exist [19], previous studies have shown an additional benefit of PTA in patients with flexed knee gait [13,14]. Stout et al. reported an increased correction of 20° when crouch gait is addressed with DFEO and PTA in comparison to DFEO alone [13]. The data of the present study showed no significant difference in the kinematic results between patients that had additional PTA and those who did not. However, it must be critically acknowledged that, due to the sample size in this study, a subgroup analysis is difficult. Only seven patients (nine limbs) received additional PTA, and a positive effect of the procedure on the mid-term outcome might not have been detectable due to the small sample size.

## 5. Conclusions

The present study indicates that DFESO improves flexed knee gait and has a lasting positive impact on sagittal plane kinematics in children with cerebral palsy and crouch gait. However, further investigations are needed to identify those patients that benefit most from the procedure and explain the detected tendency towards recurrence at mid-term follow-up. The results of the study are limited by the sample size and the retrospective study design. Furthermore, the additional procedures performed might influence the results of the study.

## Figures and Tables

**Table 1:** Overview of the retrieved data of patients included in our sample.

Age	FU	GMFCS	Side	DFESO	PTA	Bony Reconstruction Hip	Soft Tissue Surgery of the Hip	Recession or Transposition of Rectus Femoris	Hamstring Lengthening	Additional Derotational Osteotomy	Equinus Correction	Hindfoot Reconstruction
6.7	55.2	I	L	+						+	+	
			R	+						+	+	
11.6	33.6	II	L	+			+	+		+	+	+
			R	+			+	+		+	+	+
11.6	33.5	II	L	+			+	+	+	+	+	
			R	+			+	+	+	+	+	
13.8	31.8	II	L	+								+
			R	+								+
13.4	48	II	L	+						+	+	
			R								+	
12.2	26.4	II	L	+			+	+		+	+	
			R				+	+				
15.4	33.9	II	L	+			+	+			+	
			R	+			+	+			+	
13.5	47.3	II	L	+	+			+	+	+	+	
			R		+			+			+	+
11.1	24.4	II	L	+						+	+	+
			R	+						+	+	+
7.2	26.4	III	L	+			+	+	+		+	+
			R	+				+	+		+	+
8.9	25.1	III	L	+			+			+	+	
			R			+			+		+	
15.8	26.8	III	L	+	+		+			+		
			R				+		+	+		
10	54.8	II	L		+						+	+
			R	+	+					+	+	
9.2	42.3	II	L					+		+	+	
			R	+				+		+	+	
9.3	55	III	L					+		+		
			R	+				+			+	+
16.1	27.8	III	L	+	+					+	+	
			R	+	+						+	
12.3	36.1	III	L		+					+		+
			R	+	+					+		+
12.7	54.6	III	L	+	+					+	+	
			R	+	+					+	+	
14.1	31.3	III	L		+	+					+	
			R	+	+					+	+	

FU: follow-up; GMFCS: Gross Motor Function Classification System; DEFO: distal femoral extension osteotomy; PTA: patella tendon advancement. Equinus correction = lengthening of the calf muscles. Hindfoot reconstruction included arthrodesis and soft tissue procedures, e.g., muscle transposition.

**Table 2:** Overview of the kinematic parameters at the different time points; E_0_: preoperative; E_2_: 24–60 months postoperative.

Parameter	Context	E_0_	E_2_	Significance
GDI	Overall	56.3 ± 7.6 (44–76)	70.6 ± 9.8 (49–92)	*p* = 0.001
Walking speed (M/s)	Overall	0.73 ± 0.18	0.84 ± 0.19	*p* = 0.04
Min. knee flexion at initial contact	Overall	46.8° ± 11.6°	31.3° ± 9.5°	*p* < 0.001
	DFESO + PTA	52.1° ± 6.1°	35.2° ± 9.8°	*p* = 0.01
	DFESO	44.4° ± 12.8°	29.5° ± 9.0°	*p* = 0.001
Min. knee flexion in stance	Overall	34.7° ± 18.3°	20.9° ± 10.1°	*p* < 0.001
	DFESO + PTA	41.4° ± 15.5°	25.9° ± 14.2°	*p* = 0.021
	DFESO	35.9° ± 20.1°	22.7° ± 12.2°	*p* = 0.003
Peak knee flexion in swing	Overall	65.2° ± 12.1°	58.5° ± 7.9°	*p* = 0.008
	DFESO + PTA	69.6° ± 9.6°	58.6° ± 8.5°	*p* = 0.021
	DFESO	63.2° ± 12.8°	58.4° ± 7.8°	*p* = 0.12
Mean knee flexion over 100% GC	Overall	49.6° ± 14.2°	37.9° ± 9.4°	*p* < 0.001
	DFESO + PTA	56.3° ± 10.8°	39.2° ± 10.9°	*p* = 0.015
	DFESO	46.5° ± 14.7°	33.2° ± 10.8°	*p* = 0.005
Mean hip flexion in stance	Overall	26.3° ± 10.4°	18.9° ± 8.0°	*p* = 0.001
	DFESO + PTA	30.1° ± 4.6°	23.5° ± 5.5°	*p* = 0.86
	DFESO	24.5° ± 11.9°	16.8° ± 8.2°	*p* = 0,007
Mean pelvic tilt	Overall	13.5° ± 8.4°	12.9° ± 5.6°	*p* = 0.72
	DFESO + PTA	12.6° ± 4.4°	13.7° ± 8.4°	*p* = 0.59
	DFESO	13.9° ± 9.8°	12.6° ± 4.1°	*p* = 0.53

Overall: includes patients with and without PTA (n = 19, 28 limbs); DFESO + PTA (n = 7, 9 limbs); DFESO (n = 12, 19 limbs). Statistical significance was reached when *p* < 0.05.

**Table 3:** Overview of the kinematic parameters at the different time points of patients with repeated postoperative gait analysis (n = 15; 22 limbs).

Parameter	Context	E_0_	E_1_	E_2_	Significance
Min. knee flexion in stance	Overall	40.0° ± 17.9°	20.8° ± 13.1°	23.3° ± 12.8°	¶, ⎕
Mean knee flexion in stance	Overall	46.2° ± 21.1°	27.5° ± 11.3°	34.9° ± 11.8°	¶, §, ⎕
Knee flexion at initial contact	Overall	48.2° ± 11.4°	29.6° ± 10.5°	32.8° ± 8.5°	¶, ⎕
Peak knee flexion in swing	Overall	66.4° ± 12.3°	53.0° ± 11.1°	58.6° ± 8.6°	¶, §, ⎕
Mean knee flexion over 100% GC	Overall	51.0° ± 14.9°	32.8° ± 10.5°	38.0° ± 9.8°	¶, ⎕
Mean hip flexion in stance	Overall	27.3° ± 2.4°	21.3° ± 1.9°	21.2° ± 1.5°	¶, ⎕
Mean pelvic tilt	Overall	13.9° ± 9.5°	15.3° ± 9.2°	13.3° ± 6.4°	

E_0_: preoperative; E_1_: 1–18 month postoperative; E_2_: 24–60 month postoperative; ¶: significant change between E_0_ and E_2_ (*p* < 0.05); ⎕: significant change between E_0_ and E_1_; §: significant change between E_1_ and E_2_ (*p* < 0.05).

## Data Availability

All of the data analyzed in this manuscript can be provided upon request by contacting the corresponding author.
